# Mesenchymal stem cells promote pancreatic adenocarcinoma cells invasion by transforming growth factor-β1 induced epithelial-mesenchymal transition

**DOI:** 10.18632/oncotarget.9319

**Published:** 2016-05-12

**Authors:** Hai-Sen Zhou, Xiao-Fang Su, Xing-Li Fu, Guo-Zhong Wu, Kun-Lun Luo, Zheng Fang, Feng Yu, Hong Liu, Hong-Juan Hu, Liu-Sheng Chen, Bing Cai, Zhi-Qiang Tian

**Affiliations:** ^1^ Department of General Surgery, Wuxi People's Hospital, Nanjing Medical University, Wuxi 214023, P.R. China; ^2^ Nanjing Lishui People's Hospital, Nanjing 211200, P.R. China; ^3^ Department of Rehabilitation Medicine, The 101st Hospital of Chinese PLA, Wuxi 214044, P.R. China; ^4^ Health Science Center, Jiangsu University, Zhenjiang 212013, P.R. China; ^5^ Department of General Surgery, The 101st Hospital of Chinese PLA, Wuxi 214044, P.R. China

**Keywords:** mesenchymal stem cells, pancreatic adenocarcinoma, epithelial-mesenchymal transition, transforming growth factor-β1

## Abstract

Mesenchymal stem cells (MSCs) could be ideal delivery vehicles for antitumor biological agents in pancreatic adenocarcinoma (PA). While the role of MSCs in tumor growth is elusive. Inflammation is an important feature of PA. In this study, we reported that MSCs pre-stimulated with the combination of TNF-α and IFN-γ promote PA cells invasion. The invasion of PA cell lines were evaluate by wound healing assay and transwell assay *in vitro* and liver metastasis in nude mice. We observed MSCs pre-stimulated with the combination of TNF-α and IFN-γ promoted PA cells invasion *in vitro* and *in vivo*. Consistent with MSCs promoting PA cells invasion, PA cells were found undergo epithelial-mesenchymal transition (EMT). We demonstrated that MSCs pre-stimulated with both of TNF-α and IFN-γ provoked expression transforming growth factor-β1 (TGF-β1). MSCs promoting EMT-mediated PA cells invasion could be reversed by short interfering RNA of TGF-β1. Our results suggest that MSCs could promote PA cells invasion in inflammation microenvironment and should be cautious as delivery vehicles in molecular target therapy.

## INTRODUCTION

Pancreatic adenocarcinoma (PA) is an aggressive disease that develops in a fairly symptom-free manner and only 8% of patients with PA are diagnosed at the early stage [1]. Less than 20% of cases are clinically amenable to surgical resection at the time of diagnosis [2]. The five-year survival of PA is less than 5% [3], of which the mainly reasons are early metastasis and high resistance to chemotherapy [4]. Current available treatment choices have not significant improvement in overcoming the early metastasis and chemo-resistant of PA in the recent decades [5].

Increasing evidence suggest that mesenchymal stem cells (MSC) could be ideal delivery vehicles for antitumor biological agents [6, 7]. MSCs are adult stem cells that can differentiate into multiple lineages such as astroycytes, adipocytes, chondrocytes, myocytes and osteocytes [8]. MSCs are found not only in the marrow but also other sites in the body such as uterus and adipose tissue [9]. The antitumor properties that MSCs have affinity for site of tumor, immune privilege nature lead much of interest in the role of MSCs in tumors [10]. The probable tumor-specific migration of MSCs is still illegible. Since the tumors are characterized as ‘wounds that do not heal’, its microenvironment is considered as a site of chronic inflammation [11]. High concentrations of growth factors and inflammation chemokines believed to be responsible for MSCs recruitment into tumor stroma [12]. Number of antitumor genes have been engineered into MSCs for delivery to tumor stroma and successfully demonstrated antitumor effects on various tumor models. However, some studies suggest that MSCs have several tumor growth promoting effects in the tumor microenvironment [13].

Epithelial-mesenchymal transition (EMT) is a complex process involved in embryonic development, wound healing and carcinogenesis. During this process, epithelial cells lose their defining characteristics and acquire mesenchymal properties: loss of cell-cell adhesion, increased motility and invasiveness, and resistance to apoptosis and changes in cellular morphology [14]. When EMT occurs in tumor cells, the cells lose their epithelial features and acquire a more migratory and invasive phenotype leading to augmented metastatic potential. An increasing number of evidences reveal its essential role in the local progression and metastasis of pancreatic cancer [15, 16]. Transforming growth factor-β1 (TGF-β1) is one of the most important members of the transforming growth factor family. It is a potent inducer of epithelial plasticity leading to EMT in cancer cells. Increasing studies point out the importance of TGF-β1 in cancer progression and metastasis [17, 18].

In the present study, we aimed to investigate the role of MSCs on the invasion of PA cells in inflammation microenvironment and its potentially mechanism. We found that MSCs pre-stimulated with the combination of TNF-α and IFN-γ promoted PA cells invasion by TGF-β1 induced EMT.

## RESULTS

### MSCs pretreated by TNF-α and IFN-γ promotes the migration and invasion of PA cells *in vitro*

**Figure 1:**
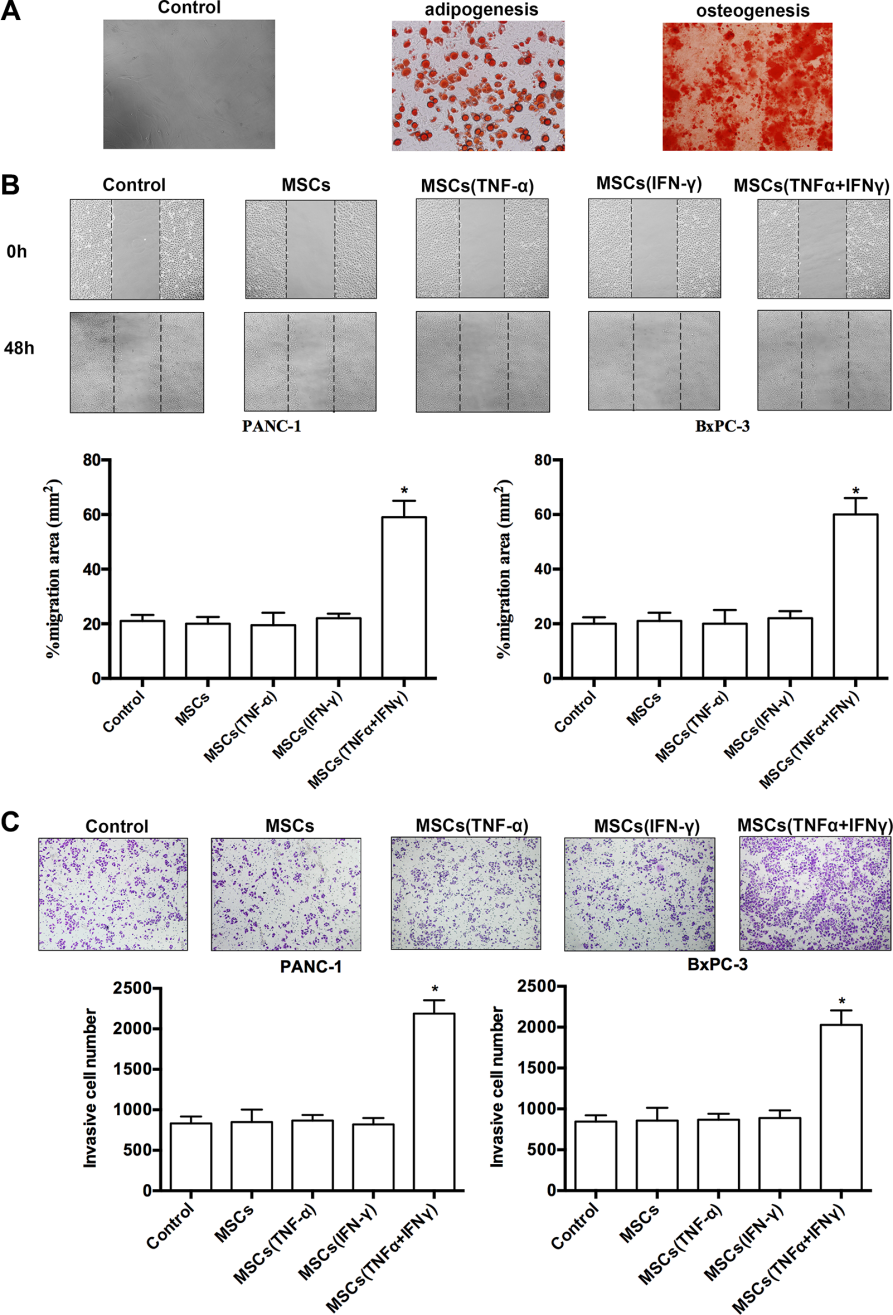
MSCs promoted pancreatic adenocarcinoma cells migration and invasion *in vitro* (**A**) MSCs were grown under different conditions that favor differentiation into either adipocytes or osteoblasts. The indicative adipocytes were triglycerides by staining with oil red O. The characteristic of osteoblasts was calcium deposition by staining with Von Kossa. (**B**) The wound healing assay was used to evaluate migration of PANC-1 and BxPC-3 cells. Photographs were taken in a phase-contrast microscope at 0 hr and 48 hr when PA cells co-cultured with ‘the conditioned medium’ of MSCs. Results of %migration area presented represent mean ± SD (*n* = 3) (*P < 0.05; 200 ×). (**C**)Invasiveness of PANC-1 and BxPC-3 cells was determined by using the Transwell assay. PA cells were cultured with ‘the conditioned medium’ of MSCs, and then plated in the upper chamber of the transwell and allowed to grow for 24 hr in serum-free medium, 5% fetal bovine serum was placed in the lower chamber. Number of cells that invaded through the matrigel was counted in 10 fields under the 200 × objective lens. Results of invasive cell number presented represent mean ± SD (*n* = 3) (*P < 0.05; 200 ×).

Human MSCs were a gift from Institute of Health Sciences and Shanghai Institute of Immunology, Chinese Academy of Sciences, Shanghai, China [19]. We firstly investigate whether the human MSCs have multi-lineage differentiation potential. As was shown in Figure 1A, the human MSCs could differentiate into adipocytes and osteoblast-like cells under different culture conditions.

To investigate the effect of MSCs on the migration and invasion of PA cells in inflammatory environment, we determined influence on motility changes of PA cell lines-PANC-1 and BxPC-3 in the presence of different types of conditioned medium from cultures of MSCs. The migratory ability of PA cells *in vitro* was evaluated by wound healing assay. The migratory ability of PA cells was obviously promoted when cells were cultured with conditioned medium of MSCs pre-stimulated with both of TNF-α and IFN-γ (Figure 1B). The enhanced migratory ability was not observed in PA cells when they were cultured with conditioned medium of MSCs alone or stimulated with TNF-α, and IFN-γ. Furthermore, PA cells invasion ability was determined by transwell assay to verify the influence of MSCs. The results confirmed that PA cells invasion ability markedly increased after co-culture conditioned medium of MSCs pre-stimulated with both of TNF-α and IFN-γ rather than control groups (Figure 1C). The results demonstrated that conditioned medium of MSCs pre-stimulated with the combination of TNF-α and IFN-γ could significantly promote the migration and invasion in PA cell lines (Figure 1B, 1C).

### Assessment of liver metastasis *in vivo*

**Figure 2:**
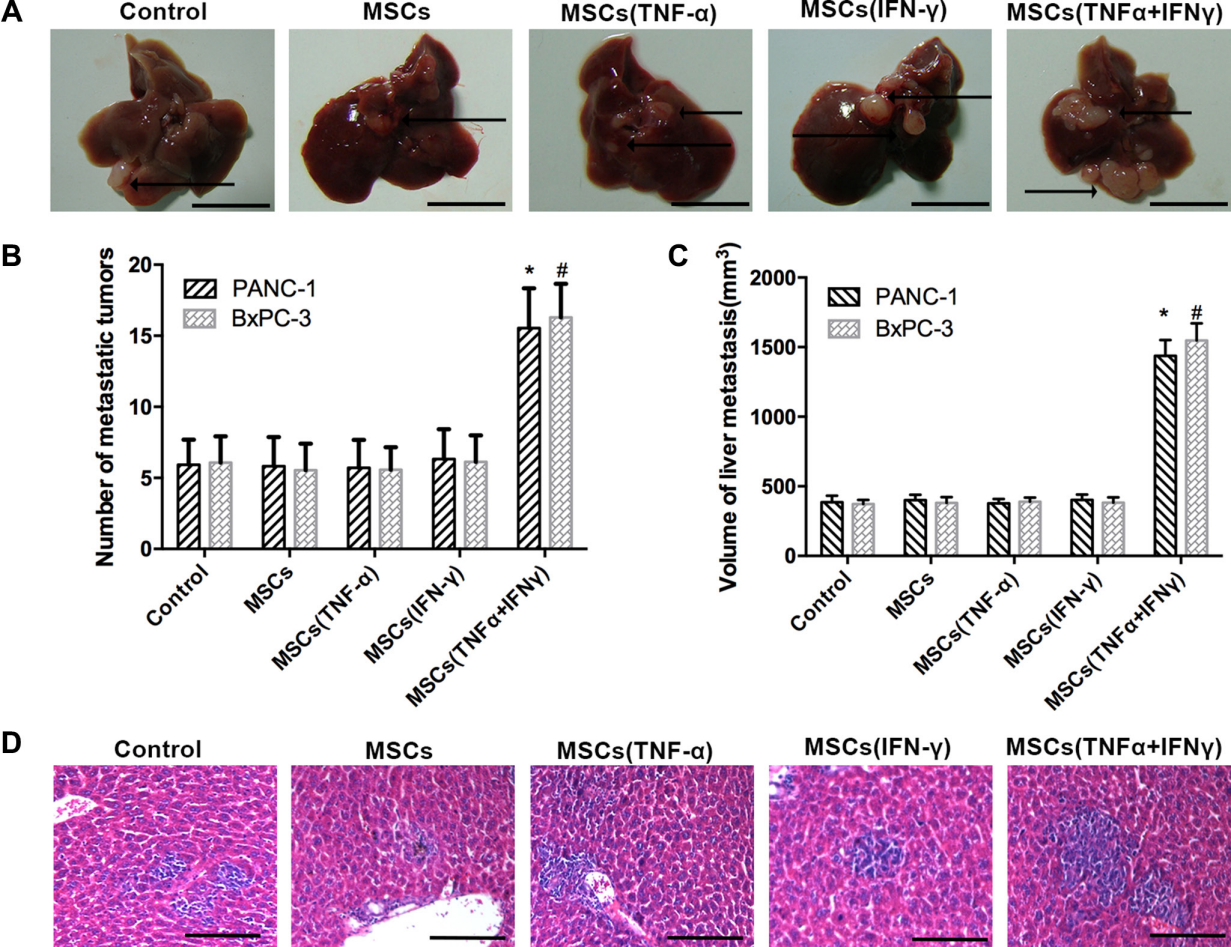
MSCs promoted liver metastasis of pancreatic adenocarcinoma in nude mice The tumor models are described in the “Materials and Methods” section. (**A**) Representative liver metastatic tumors are shown in nude mice 6 weeks after splenic injection. The arrows indicate the metastatic tumor on the surface of the liver. Scale bar, 10 mm. (**B**, **C**) The number of liver metastases and the volume of nodules were measured. (**D**) Microscopic images of livers that were resected, sectioned, and stained with H&E are shown. The arrow indicate the metastatic tumor in the liver issue. Scale bar, 100 μm. Data represent the mean of three independent experiments and were shown as mean ± SD (*P < 0.05).

To further observe the effects of MSCs on tumor metastasis of pancreatic cancer *in vivo*, we established an animal model with liver metastasis from human PA by PANC-1 and BxPC-3 cells. After 6 weeks of splenic vein co-injection PA cells with MSCs being stimulated by TNF-α and IFN-γ, experimental nude mice were sacrificed. At the same time, livers were excised and then weighed (Figure 2A). Hematoxylin and eosin (H&E) staining was performed on serial sections of metastatic tumors and normal liver (Figure 2D). It was observed that there was a dramatic increase in the number of liver metastases in mice receiving PA cells co-injection with MSCs being pre-stimulated with TNF-α and IFN-γ versus control group (Figure 2B). The same results were obtained in the volume of liver metastases (Figure 2C). All these evidences indicated that MSCs pre-stimulated with the combination of TNF-α and IFN-γ significantly promoted the progression of liver metastasis from PA.

### PA cells underwent EMT after exposed to conditioned medium of MSCs per-stimulated by TNF-α and IFN-γ

**Figure 3:**
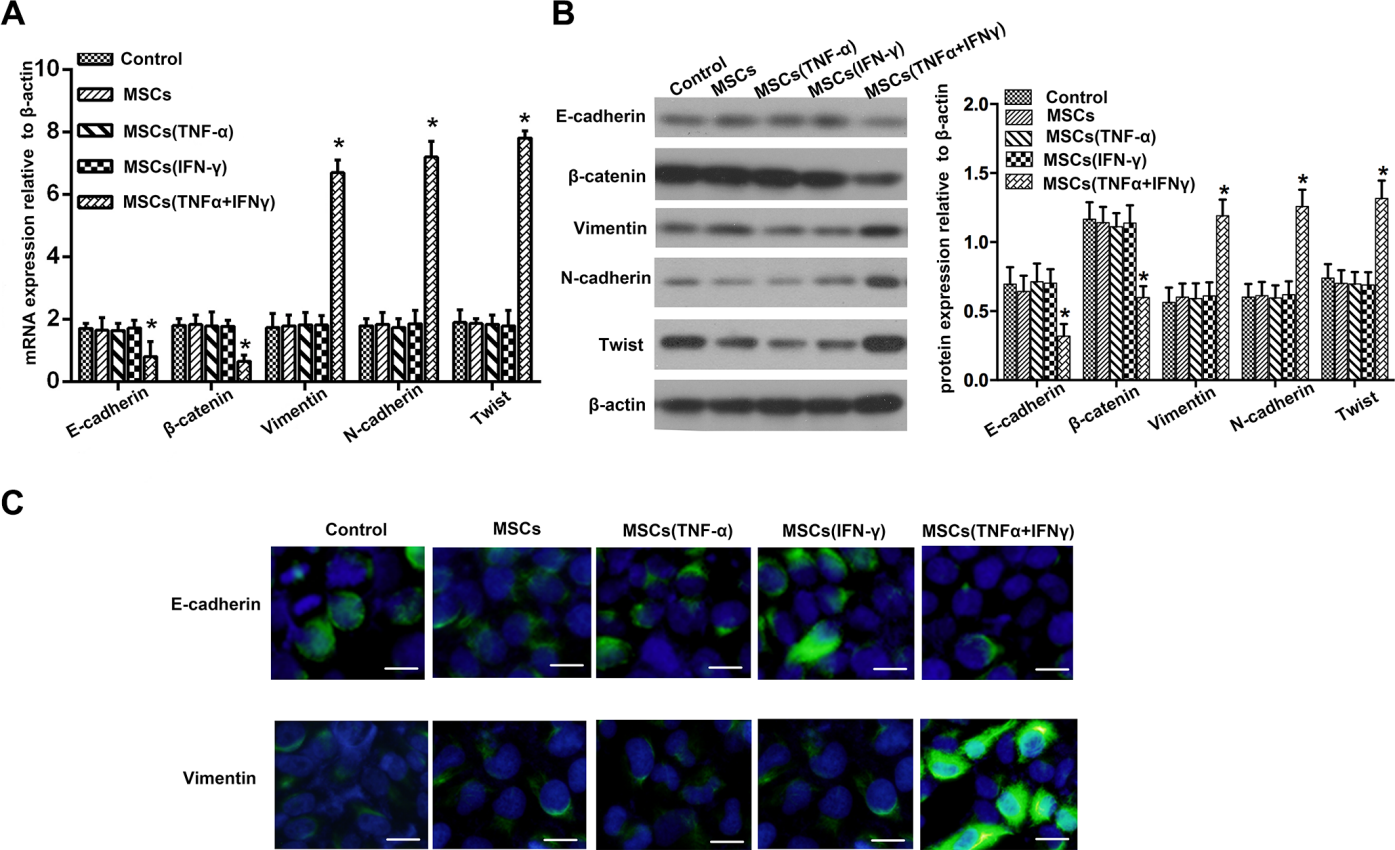
Pancreatic adenocarcinoma cells underwent EMT after co-culture with the culture supernatant of MSCs (**A**) Real-time PCR was used to detect changes in expression of EMT genes on mRNA level in PANC-1 cells following co-culture with the culture supernatant of MSCs pretreated with TNF-α and IFN-γ. Results presented represent mean ± SD (*n* = 3) (*P < 0.05). (**B**) The expression of E-cadherin, β-catenin, Vimentin, N-cadherin and Twiston protein level in PANC-1 cells were detected using Western blot. (**C**) Immunofluorescent staining of E-cadherin and Vimentin was performed in PANC-1 cells; nuclei were counterstained with DAPI (200 ×). Scale bar, 100 μm.

Because more and more evidence reveals its essential role in the local progression and metastasis of PA [15, 20], we detected the EMT markers in PANC-1 cells when they cultured with conditioned medium of MSCs pre-stimulated with TNF-α and IFN-γ. We performed real-time PCR and Western blot analysis of the expression of an epithelial marker (E-cadherin and β-catenin), mesenchymal markers (Vimentin and N-cadherin) and EMT-associated transcriptional factors (Twist). The result showed that consistent with MSCs promoting PA cells invasion in inflammation microenvironment, E-cadherin and β-catenin were down-regulated, whereas Vimentin, N-cadherin and Twist were up-regulated on mRNA and protein expression level (Figure 3A, 3B). PANC-1 cells that cultured with MSCs respectively pretreated by TNF-α and IFN-γ did not display the typical EMT markers, as well as the control group (Figure 3A, 3B). The result of E-cadherin and Vimentin immunofluorescent further supported the finding (Figure 3C). These results indicated that MSCs promoted PA cells invasion by EMT-mediated in inflammatory environment.

### TGF-β1 was up-regulated in the culture supernatant of MSCs after being stimulated by TNF-α and IFN-γ

**Figure 4:**
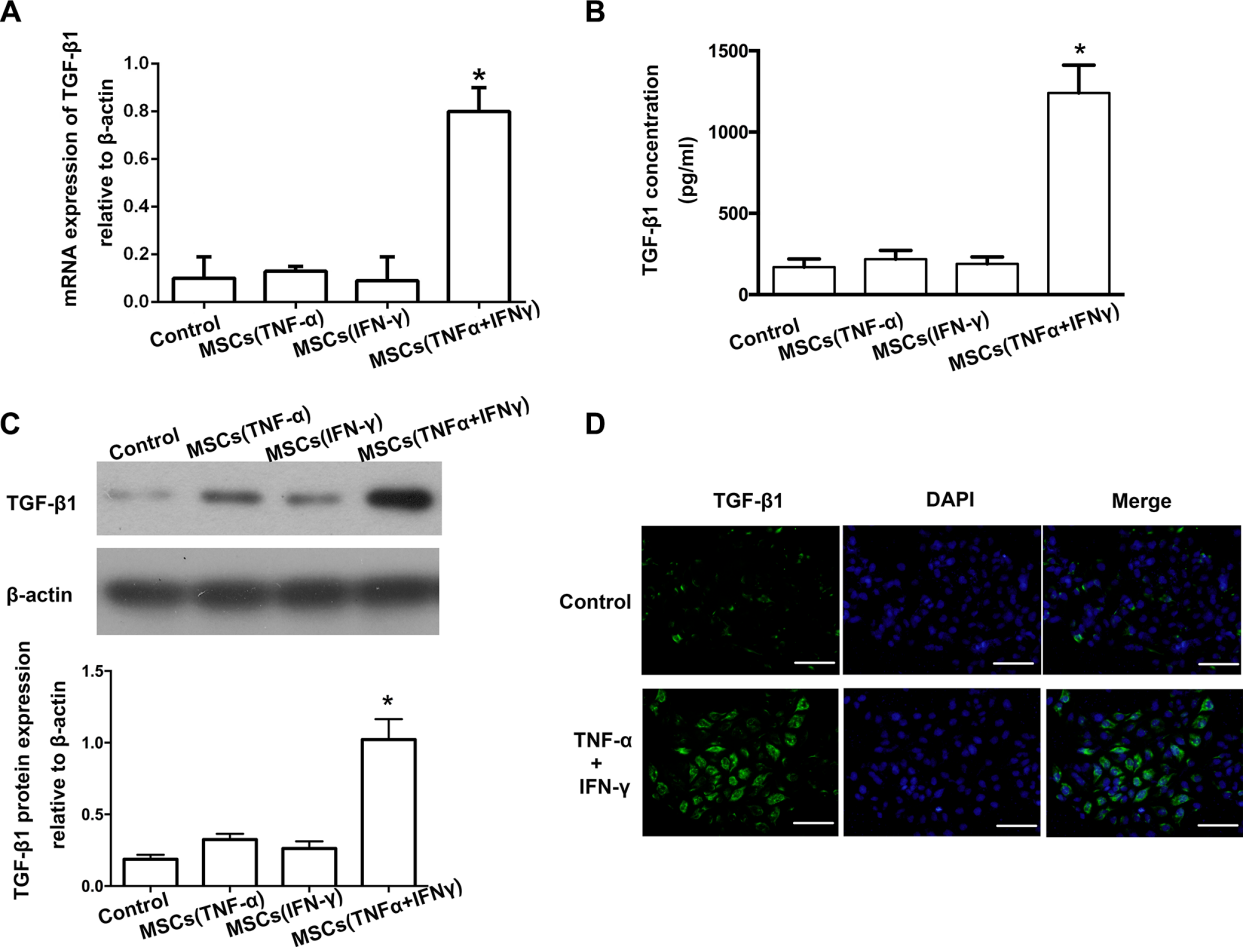
TGF-β1 was up-regulated in the culture supernatant of MSCs after being stimulated by TNF-α and IFN-γ (**A**) Real-time PCR result showed that the expression of TGF-β1 on mRNA level was increased in the culture supernatant of MSCs pre-stimulated with TNF-α and IFN-γ (*P < 0.05). (**B**) The concentration of TGF-β1 in the culture supernatant of MSCs was detected by ELISA. (**C**) Western-blot was used to detected TGF-β1 expression in the culture supernatant of MSCs, and the results showed that stimulated with both of TNF-α and IFN-γ caused a significant up-regulation of TGF-β1. (**D**) Immunofluorescent staining was used to confirm that up-regulation of TGF-β1 in MSCs pre-stimulated with TNF-α and IFN-γ (200 ×). Scale bar, 500 μm.

TGF-β1 is one of the best known inducers of EMT in cultured epithelial cells and is known to be a tumor promoter in cancers. We proved that the EMT in pancreatic cancer cells is caused by exogenous TGF-β1 secreted by MSCs. Therefore, we detected TGF-β1 expression in the culture supernatant of MSCs stimulated with the combination of TNF-α and IFN-γ. Real-time PCR and Western blot results showed that stimulation with the combination of TNF-α and IFN-γ provoked the expression of TGF-β1 at both mRNA and protein levels in the culture supernatant of MSCs (Figure 4A, 4C). TGF-β1 expression level in the culture supernatant of MSCs pretreated with both TNF-α and IFN-γ obviously was up-regulated more highly than those in the control group and pretreated separately. ELISA results also revealed that the amount of TGF-β1 in the culture supernatant of MSCs stimulated with TNF-α and IFN-γ was remarkably up-regulated than control groups (Figure 4B). Although the protein level of TGF-β1 is increased by TNF-α stimulating alone, the concentration of TGF-β1 may be still not enough to induce EMT in PA cells. The results of immunofluorescence further supported that MSCs significantly over expressed TGF-β1 when pretreated with both TNF-α and IFN-γ (Figure 4D).

### Inhibition TGF-β1 expression of MSCs reverses the promoting EMT-mediated invasion in PA cells

**Figure 5:**
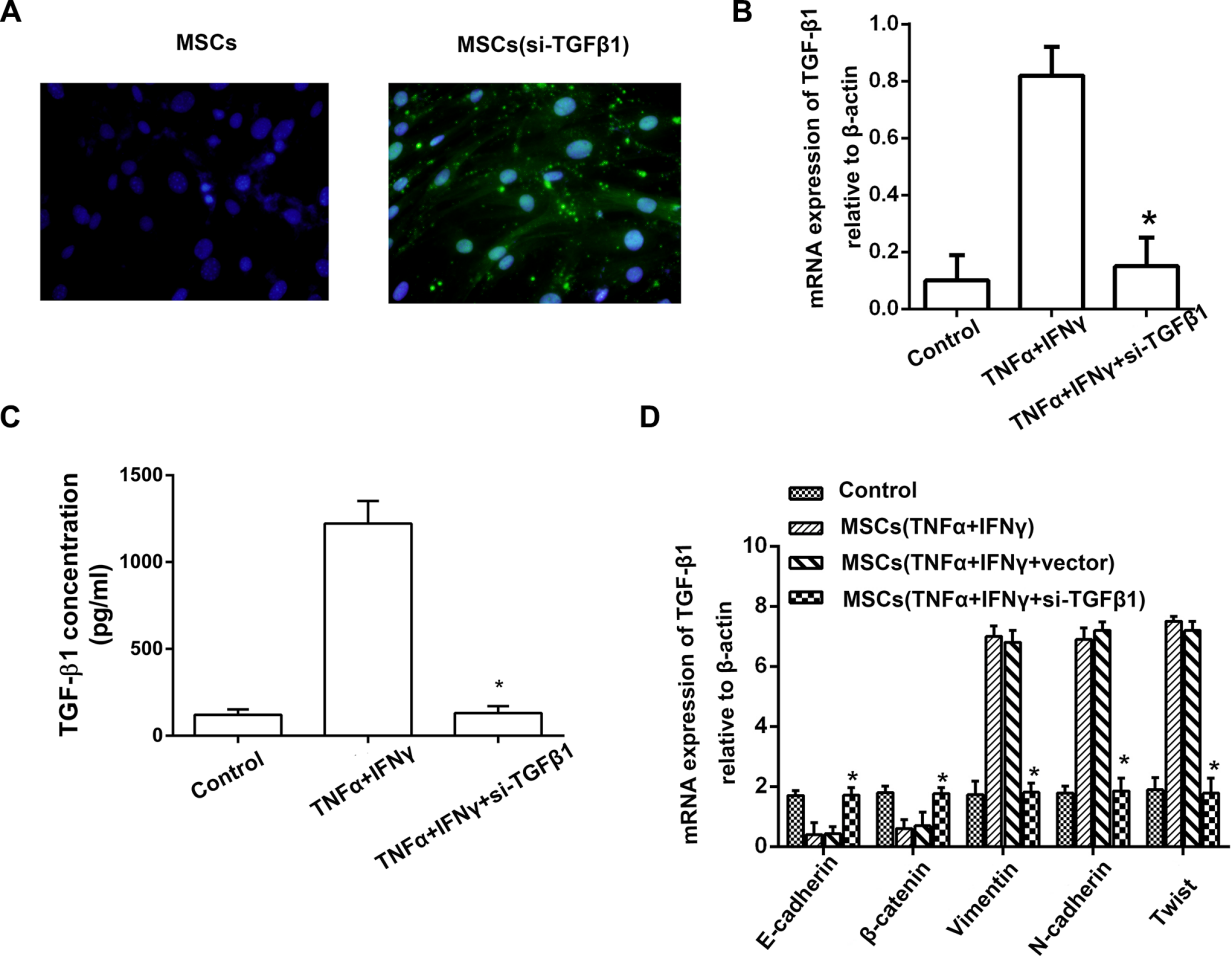
TGF-β1 knockdown of MSCs reverses the EMT in pancreatic adenocarcinoma cells (**A**) Transfections of TGF-β1 siRNA into MSCs were performed with a Lipofectamine, and FAM was observed under fluorescence microscope (200 ×). (**B**) Real-time PCR result showed that the expression of TGF-β1 in the culture supernatant of MSCs^si-TGFβ^ was markedly decreased, the inhibitory efficiency was more than 90% compared with the MSCs^vector^ (*P < 0.05). (**C**) ELISA result revealed that the amount of TGF-β1 in the culture supernatant of MSCs^si- TGF-β1^ was remarkably down-regulated even they were pretreated with both of TNF-α and IFN-γ. (**D**) PANC-1 cells cultured with the culture supernatant of MSCs^si-iNOS^, and then the expression of EMT genes on mRNA level in PANC-1 cells were detected by real-time PCR (*P < 0.05).

To investigate the role of TGF-β1 in the promoting EMT-mediated invasion of PA cells, TGF-β1 expression in MSCs was knockdown using siRNA against TGF-β1 mRNA. All the three candidate sequences could effectively inhibit TGF-β1 expression in MSCs even they were pretreated with both of TNF-α and IFN-γ (Figure 5A, 5B). ELISA results also revealed that the amount of TGF-β1 in the culture supernatant of MSCs^si- TGF-β1^ was remarkably down-regulated (Figure 5C). Then, we investigated the effect of TGF-β1 knockdown MSCs on EMT in PA cells in inflammation microenvironment. Real-time PCR results of expression of EMT genes confirmed that TGF-β1 knockdown in MSCs led to up-regulation of E-cadherin and β-catenin, and down-regulation of Vimentin, N-cadherin and Twist, compared with control groups (Figure 5D).

**Figure 6:**
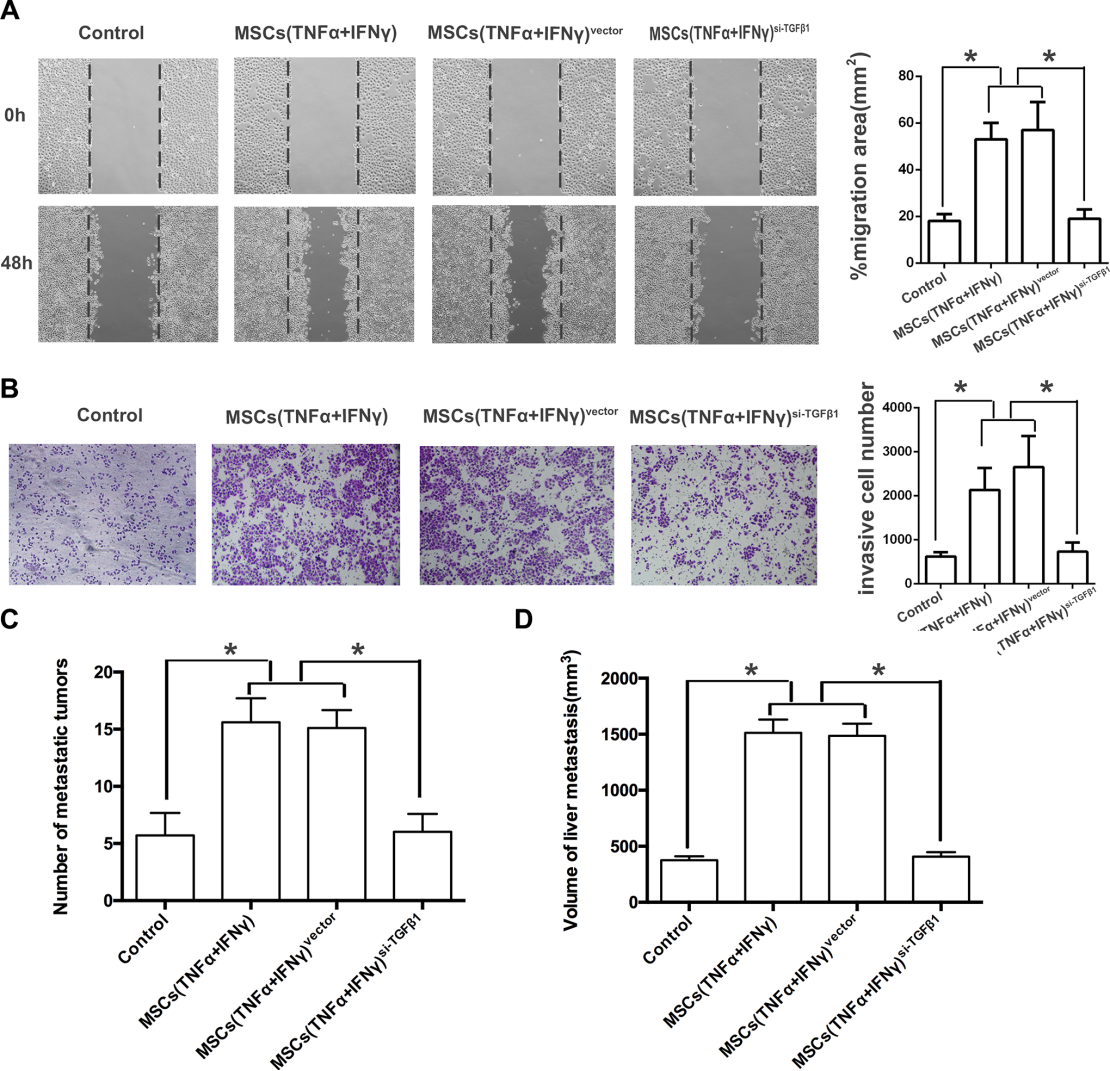
TGF-β1 knockdown of MSCs reverses the EMT in pancreatic adenocarcinoma cells (**A**) Transfections of TGF-β1 siRNA into MSCs were performed with a Lipofectamine, and FAM was observed under fluorescence microscope (200 ×). (**B**) Real-time PCR result showed that the expression of TGF-β1 in the culture supernatant of MSCs^si-TGFβ^ was markedly decreased, the inhibitory efficiency was more than 90% compared with the MSCs^vector^ (*P < 0.05). (**C**) ELISA result revealed that the amount of TGF-β1 in the culture supernatant of MSCs^si- TGF-β1^ was remarkably down-regulated even they were pretreated with both of TNF-α and IFN-γ. (**D**) PANC-1 cells cultured with the culture supernatant of MSCs^si-iNOS^, and then the expression of EMT genes on mRNA level in PANC-1 cells were detected by real-time PCR (*P < 0.05).

What changed the invasion of PA cells when cultured with TGF-β1 knockdown MSCs in inflammation microenvironment? The result of wound healing assay and transwell assay showed that the invasive ability *in vitro* was significantly reduced compared with control group (Figure 6A, 6B). The number of liver metastases as well as the volume of nodules in mice receiving PA cells co-injection with TGF-β1 knockdown MSCs was obviously reduced compared with control groups (Figure 6C, 6D). All these data supported that MSCs promoted PA cells invasion by TGF-β1 induced EMT in inflammation microenvironment.

## DISCUSSION

PA is the most aggressive cancer with a very poor prognosis in patients with this disease. Resistance to chemotherapeutic drugs and early metastasis are major cause of treatment failure [21]. The treatment of PA remains a major challenge [3]. Therefore, there is an urgent need for new therapeutic strategies for PA treatment [4]. Recently, MSCs have been used as delivery vehicles for target therapy of tumor.

MSCs have the potential of differentiation into adipocytes, chondrocytes, myotubes, neural cells, osteoblasts and hematopoietic supporting storm [8]. MSCs have been used experimentally to repair tissue in various degenerative diseases and immune disorder. Taking advantage of homing potential to the primary tumor site, MSCs could be ideal delivery vehicle for anti-tumor biological agents [6]. Number of suicide genes, cytokines, chemokines and immunostimulating have been engineered into MSCs and successfully demonstrated anti-tumor effects on various tumor models [6, 7, 22].

Recent, studies have revealed that MSCs play an important role in tumor growth and metastasis especially in the tumor microenvironment [10]. MSCs contribute to pro-tumorigenic probably due to expression of growth factors, creation of cancer storm cell niches and promotion of tumor vessel formation [23]. However, the effect of MSCs on the growth and metastasis of tumor cells is controversial due to the different treatment of MSCs. Whether MSCs pro-tumorigenic or tumorsuppressive seems to be depended upon biological properties of tumor microenvironment [24]. Inflammation is an essential part of the tumor microenvironment. Chemokines, cytokines and leukocyte infiltration are crucial elements, which contribute to tumor-related inflammation. In our study, we imitate the inflammatory tumor microenvironment by adding TNF-α and IFN-γ into the culture medium of MSCs. After pretreated with TNF-α and IFN-γ, the supernatant culture medium of MSCs was co-cultured with pancreatic cancer cells. The results demonstrated the invasion of pancreatic cancer cells was promoted by the supernatant of MSCs.

To explore the potential mechanisms involved in pro-tumorigenic of MSCs, we investigated the molecular markers for EMT in pancreatic cancer cells. EMT is traditionally believed to be capable of enhanced motility and invasive properties *in vitro* and competent for the metastatic cascade *in vivo* [25]. During the process of EMT, there are changes in expression of multiple transcription factors, surface receptors, and secreted ligands [26]. In our study, PA cells were observed undergoing EMT, which was associated with increasing motility and invasive properties.

TGF-β is one of the best known inducers of EMT in cultured epithelial cells [27]. TGF-β has potent growth inhibitory effects on non-neoplastic epithelial cells but dual role in advanced cancers [27, 28]. The effects of TGF-β depend on the cellular context, and this contextual nature is particularly manifest in cancers. TGF-β from the inflammatory tumor microenvironment may cause cancer cell apoptosis and tumor suppression [29] or induce an EMT that promotes cancer cell invasion and metastasis [30] or promote cancer stem cell heterogeneity and drug resistance [31]. David et al. [28] elucidate the dichotomous effects of TGF-β on pancreatic ductal adenocarcinoma cells, which TGF-β drives tumor suppression in pancreatic cancer cells by promoting EMT-linked remodeling of the transcription factor landscape. During recent years, TGF-β1 has been shown to play an important role in EMT [32, 33]. TGF-β1, which is expressed by tumor-infiltrating immune cells, stands out as a master regulator of the pro-invasive tumor microenvironment [34]. Thus, TGF-β1 induced EMT represents a link between cancer and inflammation [35]. In order to further investigate whether EMT of PA cells was induced by TGF-β1, we detected the expression of TGF-β1 in MSCs after being pretreated by inflammatory cytokines. As expected, pretreatment with TNF-α and IFN-γ markedly up-regulated the expression of TGF-β1 in MSCs. The promoting effect of MSCs treated with inflammatory cytokines on PA cells *in vitro* and *in vivo* could be reversed by short interfering RNA of TGF-β1. Our results elucidated that MSCs promote PA cells invasion by TGF-β1 induced EMT in inflammation tumor microenvironment.

Taken together, our study demonstrated that MSCs provoked expression TGF-β1 in inflammation microenvironment, which promoted invasive properties of pancreatic cancer cells by inducing EMT. Therefore, the study of MSCs clinical application for target therapy treating cancer needs to pay attention to tumor microenvironment. The use of MSCs as delivery vehicles in molecular target therapy should be extremely cautious, because of the tumor growth promoting effects in inflammation tumor microenvironment.

## MATERIALS AND METHODS

### Cell lines and animals

Human MSCs was a gift from Institute of Health Sciences and Shanghai Institute of Immunology, Chinese Academy of Sciences. The culture medium of MSCs was Dulbecco's modified Eagle's medium (DMEM) nutrient mix F12 with 10% fetal bovine serum (FBS) from Invitrogen Inc. Human PA cell lines-PANC-1 and BxPC-3 was obtained from Chinese Academy Cell Banks. PANC-1 and BxPC-3 cells were cultured in DMEM medium supplemented with 10% heat-inactivated FBS and l% penicillin/streptomycin. The cells were maintained in a humidified atmosphere containing 5% CO_2_ incubator at 37°C.

Male athymic BALB/c nu/nu mice (5 weeks old) were purchased from Shanghai Experimental Animal Center of the Chinese Academy of Sciences, Shanghai, China. Nude mice were housed in pathogen-free conditions. All procedures involving animals were performed in accordance with the institutional animal welfare guidelines of 101st Hospital of Chinese PLA and approved by the Ethics Committee of the 101st Hospital of Chinese PLA (Approved Code: 2015–006).

### The conditioned medium

Firstly, the culture medium was added TNF-α (20 ng/ml) and/or IFN-γ (20 ng/ml), and then MSCs were stimulated by the culture medium for 12 hr. Secondly, the culture medium of MSCs was abandoned and replaced with fresh DMEM nutrient mix F12 with 10% FBS. Thirdly, MSCs were continuing cultured in the fresh culture medium for 24 hr. Finally, the conditioned medium was collected by filtrating the supernatant culture medium of MSCs through 0.22 μm filter.

### *In vitro* wound healing assay

The method of wound healing assay has been described by Jing *et al.* [36]. Briefly, PANC-1 or BxPC-3 cells (1 × 10^5^) were seeded on 24-well dish and incubated with ‘the conditioned medium’ of MSCs for 24 hr, and then monolayer was disrupted with a cell scraper (1.2 mm width). Photographs were taken in a phase-contrast microscope at 0 hr and 48 hr. Experiments were carried out in triplicate and each point was recorded four fields.

### Invasion assay

Transwell assay was modified according to the previous description [36]. Matrigel coated Boyden chambers (8 μm pre size) at 200 μg/ml and then Boyden chamber incubated overnight. PANC-1 or BxPC-3 cells were cultured with ‘the conditioned medium’ of MSCs, and then plated in the upper chamber of the transwell and allowed to grow at 37°C for 24 hr in serum-free medium, 5% fetal bovine serum was placed in the lower chamber. The cells were fixed in 4% formaldehyde and stained with crystal violet dye. Number of cells that invaded through the matrigel was counted in 10 fields under the 200 × objective lens. Results of invasive cell number presented represent mean ± SD (*n* = 3).

### Assessment of liver metastasis *in vivo*

To establish liver metastasis of PA in nude mice, MSCs were pretreated with TNF-α and IFN-γ (20 ng/ml each) for 12 hr and then mixed with PA cells (1.5 × 10^7^ PA cells and 0.5 × 10^7^ MSCs in 0.2 ml serum-free DMEM medium). Cells were injected into the splenic vein of nude mice. Each group consisted of 8 mice. The nude mice were sacrificed 6 weeks later and the number and volume of liver metastases was calculated and statistically analyzed. Tumor volumes of metastatic tumors were measured using the following formula: length (mm) × width^2^ (mm^2^) × (π/6) [37]. Tumor tissues were then fixed, embedded in paraffin, and serially sectioned at a thickness of 4 mm. H&E staining was performed, and the sections were examined by a pathologist to verify the presence of tumors. Data represent the mean of three independent experiments and are shown as mean ± SD.

### Real-time polymerase chain reaction (PCR)

The TGF-β1 in the culture supernatant of MSCs and EMT markers in PA cells on mRNA expression level were detected by real-time PCR. The MSCs were incubated with TNF-α (20 ng/ml) and/or IFN-γ (20 ng/ml) for 12 hr, and then PA cells co-cultured with conditioned medium of MSCs. The conditioned medium of MSCs and PA cells were collected to extract the total cellular mRNA with Trizol Reagent (Invitrogen). Expression of mRNA was determined by real-time PCR using SYBR Green Master Mix (Applied Biosystems, Foster City, CA, USA). Total sample RNA was normalized to endogenous β-actin mRNA. Primers sequences using in real-time PCR were showed in Table 1. Thermocycler conditions included an initial hold at 50°C for 2 min and then 95°C for 10 min which was followed by a two-step PCR program of 95°C for 15 sec and 60°C for 60 sec repeated for 40 cycles on an Mx4000 system (Stratagene, La Jolla, CA, USA), on which data were collected and quantitatively analyzed. Expression level of mRNA is presented as fold change relative to an untreated control.

**Table 1:** Sequence of the oligonucleotides for real-time PCR

Assays	Gene	Sequence (5′ → 3′)	
Real-time PCR	N-cadherin E-cadherin Twist β-catenin Vimentin TGFβ β-actin	F R F R F R F R F R F R F R	GCGCGTGAAGGTTTGCCAGTG CCGGCGTTTCATCCATACCACAA TGAAGGTGACAGAGCCTCTGGA TGGGTGAATTCGGGCTTGTT GGTCCATGTCCGCGTCCCACTAG CGCCCCACGCCCTGTTTCTT AGCCGACACCAAGAAGCAGAGATG CGGCGCTGGGTATCCTGATGT TGGCCGACGCCATCAACACC CACCTCGACGCGGGCTTTGT GCCGAGCCCTGGACACCAAC GCGCCCGGGTTATGCTGGTT CTCCATCCTGGCCTCGCTGT GCTGTCACCTTCACCGTTCC

### Enzyme linked immunosorbent assay

ELISA assays were performed with commercial TGF-β1 ELISA kit (R&D Systems, Minneapolis, MN). MSCs were stimulated by the culture medium for 12 hr that was added TNF-α (20 ng/ml) and IFN-γ (20 ng/ml), then medium was replaced by fresh serum-free culture medium and MSCs were cultured for another 24 hr, then supernatant was collected. The amount of TGF-β1 in the culture supernatant were assayed according to the manufacturer's recommendation.

### Western blot assay

The TGF-β1 protein in the culture supernatant of MSCs and EMT related proteins in PA cells were detected by western blot assay. The method of western blot assay was performed as described [37]. The specific antibodies for either TGF-β1 (Abcam) or β-actin (lnvitrogen) and goat anti-rabbit secondary antibody (lnvitrogen) were used in western-blot experiments.

### Immunofluorescence

Firstly, the cells (1 × 10^4^) were seeded on a 48-well dish for 24 hr Secondly, the cells were washed with PBS twice, and then fixed in 4% paraformaldehyde and 0.1% Triton × 100 in PBS buffer at 40°C for 30 min Thirdly, the cells were washed with PBS, and incubated with the blocking solution (1% goat serum in PBS), and then incubated with the primary antibodies for overnight. Finally, after being washed with PBS, the cells incubated with secondary antibodies (lnvitrogen) at 37°C for 2 hr, and the stained with DAPI. We photographed all matched samples using immunofluorescence microscope and identical exposure times.

### Short interfering RNA (siRNA) synthesis and transient transfection

We designed three siRNA sequences of TGF-β1 by using Oligoengine software and confirmed by nucleotide BLAST searches. Table 2 listed the three putative candidate sequences and a scrambled sequence these were not significant homology. We used a Lipofeetamine according to the kit's instructions. Cells (1–3 × 10^6^) grew to confluency of 50%–60% in l0 cm Petri dishes, and the siRNA sequence or their relative mock sequences transfected the cells. When the cells had been transfected for 48 hr, we observed them by fluorescence microscope and then harvested the cells.

**Table 2:** Sequence of the oligonucleotides for siRNA construct-making assays

Assays	Gene	Sequence (5′ → 3′)	
TGF-β1siRNA	Sequence 1 Sequence 2 Sequence 3 Control	Sense Antisense Sense Antisense Sense Antisense Sense Antisense	GCAAGACUAUCGACAUGGATT UCCAUGUCGAUAGUCUUGCTT CACUGCAAGUGGACAUCAATT UUGAUGUCCACUUGCAGUGTT GCAUAUAUAUGUUCUUCAATT UUGAAGAACAUAUAUAUGCTT UUCUCCGAACGUGUCACGUTT ACGUGACACGUUCGGAGAATT

### Statistical analysis

All the Values presented are expressed as mean ± SD. We compared groups by analysis of variance (ANOVA) with a posteriori contrast by least significant difference. To ensure the accuracy of the experimental data, all data were from at least three independently experiments. A value of *P* < 0.05 was considered statistically significant. Data analysis was performed by the SPSS software (version 16; SPSS).

## Abbreviations

MSCs: Mesenchymal stem cells
EMT: epithelial-mesenchymal transition
TGF-β1: transforming growth factor β1
DMEM: Dulbecco's modified Eagle's medium
FBS: fetal bovine serum
PCR: Polymerase Chain Reaction
PBS: phosphate-buffered saline
siRNA: short interfering RNA.
